# A Sparse Reconstruction Algorithm for Ultrasonic Images in Nondestructive Testing

**DOI:** 10.3390/s150409324

**Published:** 2015-04-21

**Authors:** Giovanni Alfredo Guarneri, Daniel Rodrigues Pipa, Flávio Neves Junior, Lúcia Valéria Ramos de Arruda, Marcelo Victor Wüst Zibetti

**Affiliations:** Graduate School on Electrical Engineering and Applied Computer Science, Federal University of Technology—Paraná (UTFPR), Curitiba-PR 80230-901, Brazil; E-Mails: danielpipa@utfpr.edu.br (D.R.P.); neves@utfpr.edu.br (F.N.J.); lvrarruda@utfpr.edu.br (L.V.R.A.); marcelozibetti@utfpr.edu.br (M.V.W.Z.)

**Keywords:** ultrasonic imaging, image reconstruction, sparse reconstruction, nonquadratic regularization, nondestructive testing

## Abstract

Ultrasound imaging systems (UIS) are essential tools in nondestructive testing (NDT). In general, the quality of images depends on two factors: system hardware features and image reconstruction algorithms. This paper presents a new image reconstruction algorithm for ultrasonic NDT. The algorithm reconstructs images from *A-scan* signals acquired by an ultrasonic imaging system with a monostatic transducer in pulse-echo configuration. It is based on regularized least squares using a *l*_1_ regularization norm. The method is tested to reconstruct an image of a point-like reflector, using both simulated and real data. The resolution of reconstructed image is compared with four traditional ultrasonic imaging reconstruction algorithms: *B-scan*, SAFT, *ω-k* SAFT and *regularized least squares* (RLS). The method demonstrates significant resolution improvement when compared with *B-scan*—about 91% using real data. The proposed scheme also outperforms traditional algorithms in terms of signal-to-noise ratio (SNR).

## 1. Introduction

Ultrasonic imaging systems (UIS) have been widely used in recent decades for many applications. The ultrasonic energy is used because it is safe, noninvasive and can be inexpensively generated and detected [1]. Moreover, ultrasonic imaging equipments can be portable and easy to use. In medical-imaging, they are used to helping clinical diagnosis in several medicine specialties [1]. In industry, they also are an important nondestructive testing (NDT) tool for structural materials [2]. The ultrasonic waves propagate uniformly in homogeneous media. However, when they reach an inhomogeneity—discontinuity—a portion of sound energy is generally reflected. The reflected energy depends both on the physical characteristics—density and sound velocity—of propagation medium and discontinuity proprieties. The relationship between the amount of reflected energy and the incident energy is referred to as the acoustic reflectivity of the discontinuity [3]. The images provided by an UIS represents the acoustic reflectivity within an object. In order to produce these images, a set of piezoelectric transducers—it can be a single transducer with an active element, a transducer with multiple active elements (*phased array*) or several transducers with multiple active elements—sends ultrasonic pulses into the inspected object. After that, the UIS measures any reflected waves that are produced by internal discontinuities. The reflected waves are converted into electrical signals—this conversion is carried out by a set of piezoelectric reception transducers, which may be the same set of transmission transducer (pulse-echo configuration) or an independent set (*pitch-catch configuration*)—referred to as A-scan. Then, by applying an appropriate image reconstruction algorithm to one set of A-scan signals, it is possible to form an image of the internal reflectivity of the object.

There are several algorithms used for image reconstruction in ultrasonic NDT. The simplest algorithm, known as *B-scan*, forms an image as a matrix of dots—pixels—, wherein each column represents the spatial position of the transducer. Each row corresponds to the propagation time of the ultrasonic wave from the transducer to a defined depth in the inspected object. The intensity of each pixel is proportional to the amplitude of the *A-scan* signal related to the position of the transducer and to the propagation time. A *B-scan* image shows a profile representation—cross-section—of the inspected object. Although the B-scan reconstruction algorithm is quite simple and fast, it suffers from low lateral resolution. Additionally, transducer diameter and depth of the object may negatively affect image quality [4,5] due to beam spreading and diffraction [6]. In the early 1970s, the *synthetic aperture focusing technique* (SAFT) [7–9] was developed inspired in concepts of synthetic aperture (SA) used in airborne radar mapping systems [10]. This algorithm improves the lateral resolution of reconstructed images. In general, SAFT is implemented by delay-and-sum (DAS) operations directly on the *A-scan* signals [11–13], though it can also be implemented as a matrix-vector multiplication [14] or through Stolt migration—*ω*-*k* algorithm—[15,16] applied to the *A-scan* signals in frequency-domain [17–19]. In a recent approach, SAFT is implemented in a graphics processing unit (GPU) [20].

The B-scan and SAFT algorithms were initially developed for ultrasound imaging systems with monostatic transducers in pulse-echo configuration. Recently, however, there has been a significant increase in the UISs that use transducers with multiple elements—referred to as *phased arrays* [21]. With these transducers, UISs have the flexibility to electronically control steering, aperture and focus of the ultrasonic beam onto the discontinuities [22]. They also allow emulating the behavior of a monostatic transducer by sequentially firing each element of the array. This provides two advantages: (I) avoids physical movements of the transducer to sweep the region of interest; and (II) allows the acquisition of *A-scan* from all combinations of transmitters and receivers elements. This acquisition mode is referred to as *full matrix capture* (FMC) [21]. FMC allows other reconstruction algorithms such as *total focusing method* (TFM) [21], *inverse wave field extrapolation* (IWEX) [23], a version of *ω-k* algorithm for FMC [24], a reversible back-propagation imaging algorithm [25] and an adaptive beamforming algorithm [26]. With those algorithms, the image resolution of a point-like reflector is improved when compared with *B-scan* and SAFT [21,26,27]. Despite the advantages of phased arrays, UISs with a monostatic transducer are still widely used, especially in hand-held and embedded systems. In this configuration, only one input-output channel is required. This feature allows the design of a portable, low-cost and low-power consumption equipment, which is suitable for embedded systems [20].

This paper aims to present a new image reconstruction algorithm for NDT. The image is reconstructed from *A-scan* signals collected by an UIS with a single monostatic transducer in pulse-echo configuration. The image resolution of a point-like reflector reconstructed by the proposed method is finer than traditional image reconstruction algorithms and even FCM. In the proposed algorithm, the image reconstruction is carried out by solving a regularized least squares problem with *l*_1_ norm in regularization term that promotes a sparse reconstruction of the image [28]. This mixed-norm optimization problem is solved by the *iteratively reweighted least squares* (IRLS) and *conjugate gradient* (CG) algorithms. Although this approach has already been used in other applications such as image deblurring, tomographic reconstruction and compressed sensing [29], this is the first work, to the best of our knowledge, that uses these techniques in the context of images reconstruction for ultrasonic NDT. The remaining of the paper is organized as follows: Section 2 describes and presents an analytical model of the UIS used in the experiments. Section 3 presents the image reconstruction problem, the solution proposed and its implementation. Sections 4 and 5 show the results and discussions from both simulated and experimental data. Finally, conclusions are presented in Section 6.

## 2. Ultrasonic Imaging System

### 2.1. Description

**Figure 1:**
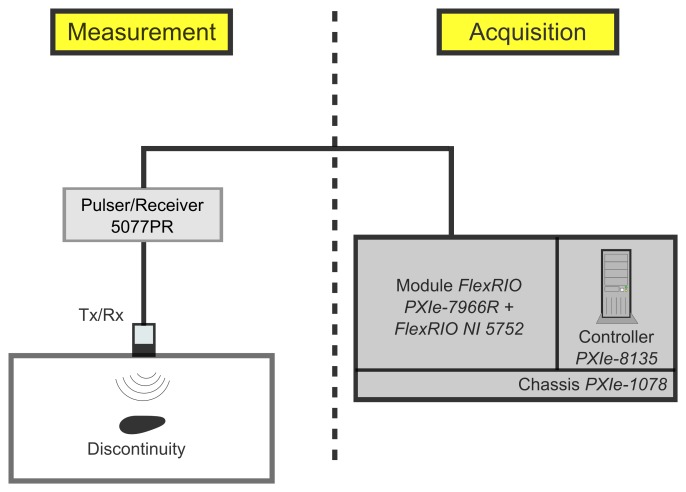
Block diagram of the UIS used in this work.

Figure 1 shows a block diagram of the UIS used in this work. The contact transducer is a *VideoScan V110-RM* produced by *Olympus*. Its active element has a circular shape with 6 mm of diameter and nominal frequency of 5 MHz [30]. It emits longitudinal ultrasonic waves. It is excited by an ultrasonic pulser/receiver model *5077PR* from *Panametrics*. The transducer is placed in direct contact with the inspected object at a normal angle to the inspection surface. The coupling material is liquid glycerin. The *A-scan* signal received by transducer is amplified in the pulser/receiver and it is digitalized by an acquisition system. This acquisition system—produced by *NI*—consists of a *PXIe-1078* chassis, a *PXIe-8135* controller and a *PXIe-7966R FlexRIO* controller with a *FlexRIO NI 5752* input and output module. The acquisition rate of *A-scan* signals is 50 MS/s and the digitized values have resolution of 12 bits. The transducer is moved laterally along the object surface by a mechanical system. The active element of the mechanical system is a servomotor—model *302865*—*driven by a EPOS2* controller, both from *Maxon*.

### 2.2. Data Acquisition

**Figure 2:**
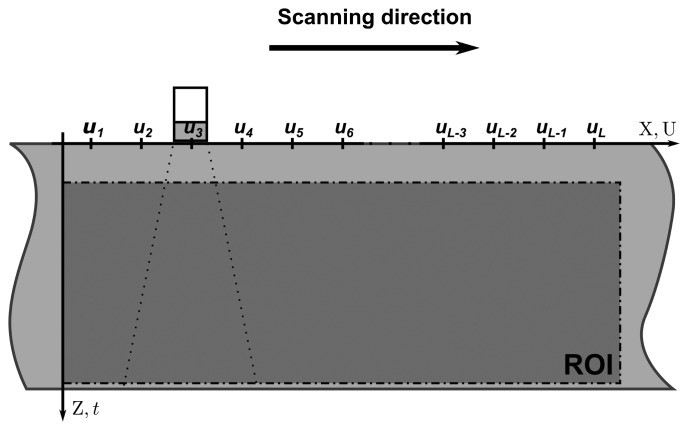
Example of a linear scan and the delimitation of the ROI in an object. The scanning direction is represented by the U axis, which coincides with the X axis of the object. The Z axis of the object—depth—is coincident with the t axis of the A-scan signals.

The *A-scan* signals acquisition process is carried out as a linear scan, wherein the transducer is placed through a set of *L* different positions along a direction—from *u*_1_ to *u_L_*. Figure 2 illustrates this process. The spatial sampling period must be constant. At each position, the pulser triggers an electric pulse and the controller acquires an *A-scan* signal. As a result, a set of *L A*-*scan* signals with *N* samples is acquired. Each one is identified by *υ_o_*(*u_l_*, *t*). The collected data is limited to a region of the object. Therefore, the reconstructed image is also limited to this region, which is referred to as *region of interest* (ROI).

### 2.3. Analytic Model

The UIS shown in Figure 1 can be modeled as a linear time invariant (LTI) system, wherein the input *υ_i_*(*t*) is the electrical signal that controls the triggering of pulser. The output *υ_o_*(*t*) is the *A*-*scan* signal [3]. As any LTI system, the output signal is related to the input signal by the convolution integral with the unit impulse response of the system *h*(*t*) [31]:

(1) $$\upsilon_{o}\left(t\right)=\int_{-\infty }^{+\infty }h\left(\tau \right)\cdot \upsilon_{i}\left(t-\tau \right)d\tau$$

Converting Equation (1) to frequency-domain and using the convolution property of Fourier transform [31], we have:

(2) $$V_{o}\left(\omega \right)=H\left(\omega \right)V_{i}\left(\omega \right)$$

where *H*(*ω*) is the *frequency response* of the system. According Schmerr, *H*(*ω*) can be represented by a combination of several cascaded LTI systems, each one representing a part of the UIS [3]. Thereby,

(3) $$H\left(\omega \right)=H_{e}\left(\omega \right)P\left(\omega \right)M\left(\omega \right)C_{T}\left(\omega \right)T_{1}\left(\omega \right)T_{2}\left(\omega \right)C_{R}\left(\omega \right)S_{A}\left(\omega \right)$$

where—for a given UIS in an experimental arrangement—*H_e_*(*ω*) is the combined frequency response of all electrical circuits—pulser, receiver and cables—, plus electrical-mechanical convertion in transducers; *P*(*ω*) is the frequency response concerning the propagation of ultrasonic waves from transmitter to receiver, through the object; *M*(*ω*) is the amplitude attenuation of the ultrasonic waves during propagation; *C_T_*(*ω*) and *C_R_*(*ω*) are the diffraction effects in ultrasonic waves due to geometry of transducers; *T*_1_(*ω*) and *T*_2_(*ω*) are the effects of water-solid interfaces—only for immersion tests—and *S_A_*(*ω*) is the scattering amplitude caused by interaction between waves ultrasonic incidents and discontinuities.

Despite the effects of some elements are not linear, linearization is possible when some conditions are assumed [3]. Regarding to propagation frequency response, these conditions are: (**I**) the UIS is in pulse-echo configuration; (**II**) the object is made of homogeneous material, with *c* referred to as the sound speed of longitudinal waves; and (**III**) the ROI is located in far field of the transducer, where the ultrasonic waves behave as plane waves. In this case, if we assume that the central point of the active surface of transducer is at position **ũ** and there is a reflector at **r̃**, then

(4) $$P\left(\omega \right)=\exp(\text{i}2\frac{\omega }{c}\left|\tilde{\text{r}}-\tilde{\text{u}}\right|)$$

The term *ω*/*c* is the spacial frequency or *wavenumber* and hereafter it is represented by *k*. As the transducer is circular, with *a* referred to as the active element radius and the ROI being in the far field, the diffraction correction factors in both transmission and reception are approximated by [3]:

(5a) $$C_{T}\left(\omega \right)=-\text{i}ka^{2}\text{jinc}(\text{ka}\sin\theta_{\tilde{r};\tilde{u}})$$

(5b) $$C_{R}\left(\omega \right)=\frac{2\pi }{-\text{i}k\pi a^{2}}C_{T}\left(\omega \right)$$

where jinc(*x*) = J_1_(*x*)/*x*, J_1_(·) is a first order Bessel function [3] and *θ***_r̃_**_;_**_ũ_** is the angle between normal axis of transducer surface and the vector **r̃** − **ũ**.

The terms *M*(*ω*), *T*_1,2_(*ω*) and *S_A_*(*ω*) of Equation (3) are approximated to the unitary constant. The attenuation of the ultrasonic waves is compensated by a variable gain amplifier in receiver circuit [1]. This amplifier has its gain controlled over time and its operation is adjusted during system calibration. There is not effects of water-solid interface because the transducer is on direct contact with the inspected object [3]. Finally, the scattering amplitude for a point-like reflector is constant by the Kirchhoff approximation [32]. Thus, replacing Equations (3)–(5) in Equation (2), and assuming that the input signal of the pulser is a high-voltage pulse with a narrow width, like a unit impulse, then *V_i_*(*ω*) can be approximated by a constant—assuming the unitary value. Thus, the frequency-domain equation that models *A-scan* signals captured by the UIS is:

(6) $$V_{o}(\tilde{u},\omega )=\left(-2a^{2}\text{i}k\right)H_{e}\left(\omega \right){\text{jinc}}^{2}(\text{ka}\sin\theta_{\tilde{r};\tilde{u}})\exp(\text{i}2k|\tilde{\text{r}}-\tilde{\text{u}}|)$$

In Equation (6) only the contribution of a point-like reflector located at position **r̂** in the signal *V_o_*(**ũ**, *ω*) is represented. Since the system is linear, the contribution of all point reflectors throughout the ROI is [28]:

(7) $$V_{o}(\tilde{u},\omega )=\left(-2a^{2}\text{i}k\right)H_{e}\left(\omega \right)\int_{\tilde{r}\in \text{ROI}}{\text{jinc}}^{2}\left(\text{ka}\sin\theta_{\tilde{r};\tilde{u}}\right)\exp\left(\text{i}2k|\tilde{r}-\tilde{u}|\right)f\left(\tilde{r}\right)d\tilde{r}$$

where *f*(**r̃**) is a function that represents the “image” of acoustic reflectivity of ROI. The function definition of *f*(**r̃**) does not indicate whether it is continuous or discrete. This depends on the definition of the ROI. In this study, both ROI as *f*(**r̃**) are discrete. Therefore, the integral in Equation (7) is replaced by a summation. The transducer moving is limited to a straight line on the surface of the inspected object, **ũ** = (*u*, 0, 0) where *u* ∈ {*u_l_* | *l* = 1, 2, …*L*}. Thus, the ROI has two-dimension (2-D) corresponding to a lateral cross section in the plane X-Z of the object. Therefore, **r̃** = (*x*, 0, *z*) where *x* ∈ {*x_l_*} = {*u_l_*} and *z* ∈ {*z_n_* | *n* = 1, 2, …*N*}. The discretized version of Equation (7) for 2-D geometry is:

(8) $$V_{o}\left(u_{l},\omega \right)=\left(-2a^{2}\text{i}k\right)H_{e}\left(\omega \right){\text{jinc}}^{2}\left(k_{u}a/2\right)\sum_{l=1}^{L}\sum_{n=1}^{N}\exp\left(\text{i}2k\sqrt{{\left(x_{l}-u_{l}\right)}^{2}+z_{n}^{2}}\right)f\left(x_{l},z_{n}\right)$$

where *k_u_* = 2*k*sin*θ*_(_*_x_l__*_,_ *_z_n__*_);_*_u_l__* is the wavenumber on acquisition axis direction (U) [16]. Applying the Fourier transform with respect to variable *u* in Equation (8), we have:

(9) $$F_{u}\left\{V_{o}\left(u_{l},\omega \right)\right\}=V_{o}\left(k_{u},\omega \right)=\left(-2a^{2}\text{i}k\right)H_{e}\left(\omega \right){\text{jinc}}^{2}\left(k_{u}a/2\right)\times \sum_{l=1}^{L}\sum_{n=1}^{N}F_{u}\left\{\exp\left(\text{i}2k\sqrt{{\left(x_{l}-u_{l}\right)}^{2}+z_{n}^{2}}\right)\right\}f\left(x_{l},z_{n}\right)$$

But according to Gough and Hawkins [16],

(10) $$F_{u}\left\{\text{exp}\left(\text{i}2k\sqrt{{\left(x_{l}-u_{l}\right)}^{2}+z^{2}}\right)\right\}=\text{exp}\left(ixk_{u}+iz\sqrt{4k^{2}-k_{u}^{2}}\right)$$

so:

(11) $$V_{o}\left(k_{u},\omega \right)=\left(-2a^{2}\text{i}k\right)H_{e}\left(\omega \right){\text{jinc}}^{2}\left(k_{u}a/2\right)\sum_{l=1}^{L}\sum_{n=1}^{N}\exp\left(ix_{l}k_{u}+iz_{n}\sqrt{4k^{2}-k_{u}^{2}}\right)f\left(x_{l},z_{n}\right)$$

However, if we define the following change of variables in the exponential term of Equation (11) [16,19]:

(12) $$\begin{array}{ll} k_{x} & \equiv k_{u}\\ k_{z} & \equiv \sqrt{4k^{2}-k_{u}^{2}} \end{array}$$

we have:

(13) $$V_{o}\left(k_{u},\omega \right)=\left(-2a^{2}\text{i}k\right)H_{e}\left(\omega \right){\text{jinc}}^{2}\left(k_{u}a/2\right)\underset{F\left(k_{x},k_{z}\right)=F_{x_{l},z_{n}}\left\{f\left(x_{l},z_{n}\right)\right\}}{\underset{︸}{\sum_{l=1}^{L}\sum_{n=1}^{N}\exp\left(ix_{l}k_{x}+iz_{n}k_{z}\right)f\left(x_{l},z_{n}\right)}}$$

Such that the highlighted sum becomes the 2-D discrete Fourier transform of function *f*(*x_l_*, *z_n_*) [33], represented by *F*(*k_x_*, *k_z_*).

The change of variables defined in Equation (12) is called *Stolt Migration* and it was initially used in processing of seismic signals [15]. This transformation makes data mapping from (*k_u_*, *ω*) domain to (*k_x_*, *k_z_*) domain [16]. Other mapping is possible and it is called *Stolt Modeling* [34]. Stolt migration and modeling are linear adjoint operators [34] and they were represented by 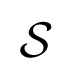 {·} and 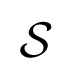
^†^ {·}, respectively. Therefore, the equation that models the UIS in spatial-temporal frequencies domain is:

(14) $$V_{o}\left(k_{u},\omega \right)=\left(-2a^{2}\text{i}k\right)H_{e}\left(\omega \right){\text{jinc}}^{2}\left(k_{u}a/2\right)S^{†}\left\{F\left(k_{x},k_{z}\right)\right\}$$

and in space-time domain is:

(15) $$\upsilon_{o}\left(u_{l},t\right)=F_{u_{l},t}^{-1}\left\{\left(-2a^{2}\text{i}k\right)H_{e}\left(\omega \right){\text{jinc}}^{2}\left(k_{u}a/2\right)S^{†}\left\{F_{x_{l},z_{n}}\left\{f\left(x_{l},x_{n}\right)\right\}\right\}\right\}$$

#### 2.4. Matrix Model

Equation (15) describes a linear model that maps an input matrix *f*(*x_l_*, *z_n_*) to an output matrix *υ_o_*(*u_l_*, *t*). By rearranging matrices *f*(*x_l_*, *z_n_*) and *υ_o_*(*u_l_,t*) as column vectors, matrices columns can be stacked as $f=\left[f_{x_{1}}^{T}f_{x_{2}}^{T}\cdots f_{x_{L}}^{T}\right]$ and $v={\left[v_{o_{u_{1}}}^{T}v_{o_{u_{2}}}^{T}\cdots v_{o_{u_{l}}}^{T}\right]}^{T}$. Thus, the matrix representation of Equation (15) becomes:

(16) v=Hf

where **H** is a matrix that models the system. It is defined as:

(17) $$H=F^{†}BS^{†}F$$

where **F** is the matrix representation of 2-D discrete Fourier transform—the direct and inverse Fourier transforms are adjoint operators, then **F**^†^ ≡ **F**^−1^ [34], **S**^†^ is the matrix representation of Stolt modeling and **B** = diag [(−2*a*^2^i*k*)*H_e_*(*ω*) jinc^2^(*k_u_a*/2)]. Uncertainties in the measurements during data acquisition process by UIS are considered into the model by adding a vector *η*. This vector contains random values, according to a Gaussian probability distribution with null mean and variance proportional to signal-noise ratio (SNR) of measurement system—Gaussian white noise. Thus, the model of the UIS in matrix form is:

(18) $$v=F^{†}BS^{†}Ff+\eta$$

## 3. Image Reconstruction Problem

Equation (18) allows simulating *A-scan* signals—vector **v**—at each transducer position *u_l_*, provided that the UIS impulse response (matrix **H**) and the acoustic reflectivity of ROI points (vector **f**) are known. When **v** is available from measurement and one wishes to estimate **f**, the problem is known as *image reconstruction problem* [35]. The simple matrix inversion **f** = **H**^−1^**v** is usually not possible due to the Hadamard conditions: (**I**) the problem may not have a solution; (**II**) it may have infinite solutions; (**III**) it may have an unstable solution. If any of those conditions is present, the problem is termed *ill-posed* [35].

### 3.1. Least Squares and Generalized Solutions

The usual approach to solve an ill-posed problem is to treat it as a minimization problem where we are looking for the vector **f̂** such as **Hf̂** is “closest” to measured signals **v**. The “closeness” between two vectors **d**_1_ and **d**_2_ is calculated by ‖**d**_1_ − **d**_2_‖*_p_*, where ‖ · ‖*_p_* represents the *l*_p_ norm vector. It is defined by [36]:

(19) $${\left\|\text{d}\right\|}_{p}={\left[\sum_{j=1}^{J}{\left|d_{j}\right|}^{p}\right]}^{\frac{1}{p}}$$

The minimization problem can be described as:

(20) $$\hat{\text{f}}=\arg\underset{\text{f}}{\min}{\left\|v-\text{Hf}\right\|}_{p}^{p}$$

where ${\left\|v-\mathrm{Hf}\right\|}_{p}^{p}$ is the *cost function* of problem. When using the *l*_2_ norm in the cost function of Equation (20), *i.e.*, *p* =2, the problem is a *least squares* (LS) and it is solved directly by [36]:

(21) $${\hat{\text{f}}}_{LS}={\left({\text{H}}^{†}\text{H}\right)}^{-1}{\text{H}}^{†}\text{v}$$

where [·]^†^ represents the adjoint operator of a matrix. For complex matrices the adjoint operator is the complex conjugate of transpose matrix, *i.e.*, **H**^†^ = [**H***^T^*]*. For real matrices adjoint and transpose are synonyms [36].

The LS method fixes the first condition of Hadamard, because there always is a found solution at least. However, to fix the second Hadamard condition a single solution must be select from a set of LS solutions [35]. The typical method used in this selection is to get a LS solution that has the lowest norm. This is done by including a constraint into the minimization problem in Equation (20) [37]:

(22) $$\begin{array}{ccc} {\hat{f}}_{MN} & =\arg\underset{\text{f}}{\min} & {\left\|\text{f}\right\|}_{2}^{2}\\ & \;\text{subject to} & \text{Hf}=\text{v} \end{array}$$

The solution for this new problem is termed *minimum norm* (MN) of a linear system or *generalized solution* [35,36] and it is solved directly by [37]:

(23) $${\hat{\text{f}}}_{\text{MN}}={\text{H}}^{†}{\left(\text{H}{\text{H}}^{†}\right)}^{-1}\text{v}$$

### 3.2. Regularization

Despite the generalized solution fixes the first two Hadamard conditions, if the matrix **H** is ill-conditioned, the generalized solution is unstable in the presence of noise. A matrix is ill-conditioned when the ratio between the largest and the smallest singular values of the matrix is very large. The singular values characterize a matrix and they are obtained by *singular value decomposition* (SVD) [38]. Singular values near zero cause noise amplification [35]. To circumvent this problem, a *regularization* technique can be used. It corresponds to include any prior information about the desired solution, to stabilize the problem and obtain a useful and stable solution [38]. The most generic form of regularization is adding a term in the cost function of Equation (20). This new term that indicates a “measure” of the solution [38] as:

(24) $$\hat{\text{f}}\left(\alpha \right)=\arg\underset{\text{f}}{\min}\left\{{\left\|\text{v}-\text{Hf}\right\|}_{p}^{p}+\alpha^{2}{\left\|\text{f}\right\|}_{q}^{q}\right\}$$

The first term of the cost function ensures fidelity of the solution with measured data. Meanwhile, the second term—referred to as *regularizer* or regularization term—inserts prior information to solution stability. The regularization parameter *α* controls the tradeoff between the two terms [35].

One of the most widely referenced regularization technique is the Tikhonov method [35]. It inserts to the original LS problem a regularization term ${\left\|\text{Lf}\right\|}_{2}^{2}$. In general, the matrix **L** is chosen as a derivative or gradient operator, so that the regularization term indicates a measure of the variability or “roughness” of image **f** [35]. The final solution is also known as *regularized least squares* (RLS). As LS, RLS also has a closed form [35]:

(25) $${\hat{\text{f}}}_{\text{RLS}}={\left({\text{H}}^{†}\text{H}+\alpha^{2}{\text{L}}^{†}\text{L}\right)}^{-1}{\text{H}}^{†}\text{v}$$

This regularization method is used by Shieh *et al.* to improve the resolution of *B-scan* images [4]. In addition to the Tikhonov method, there are other regularization techniques, formulated from different approaches. Among them, we can cite the maximum *a posteriori* (MAP) method [35] and the minimum mean square error (MMSE) method [14,39,40] both based on the Bayesian statistical approach.

More recently nonquadratic regularization methods have emerged, where the regularization term is not calculated from a *l*_2_ norm. Some of these methods are the maximum entropy [41], *total variation* (TV) [42] and nonquadratic norms [28,43]. The latter uses *l_q_* norms with *q* < 2, which does not penalize large values of their argument and the norm continues to be a convex function [35]. In Lavarello *et al.* [28] is presented a generalized Tikhonov regularization method in which the norm of regularizer is *l_q_* with *q* ≤ 1. However, when the sought solution **f** has few nonzero elements—sparse solution—*l*_1_ norm is more suitable [44,45].

In this work we used the generalized Tikhonov regularization method, such as the one used by Lavarello *et al.* with *l*_1_ norm in regularization term and **L** = **I**. Thus, the optimization problem of image reconstruction algorithm is defined as:

(26) $$\hat{\text{f}}=\arg\underset{\text{f}}{\min}\left\{{\left\|\text{v}-\text{Hf}\right\|}_{2}^{2}+\alpha^{2}{\left\|\text{f}\right\|}_{1}\right\}$$

The choice of *l*_1_ norm in regularization term was due to prior information that **f̂** should be sparse, since discontinuities in the object are very small, near to point-like reflectors. The desired image should have the lowest possible quantity of nonzero elements. The *l*_0_ norm counts nonzero elements in a vector [45], although it is nonconvex. The *l*_1_ is in some sense the convex relaxation of the *l*_0_ norm [44], keeping the convexity of Equation (26). The choice of **L** depends on what should be sparse. When **L** = **I**, the regularization term simply forces the image to be sparse. When **L** is the gradient operator (TV regularization), which accentuates the edges, the image tends to be piece-wise constant, with *sparse edges* [42]. Since we are searching for point-like reflectors, the choice **L** = **I** is more reasonable. Henceforth, this image reconstruction method is referred to as *ultrasonic sparse reconstruction* (UTSR) algorithm.

### 3.3. Algorithm Implementation

There exists a myriad of algorithms to solve quadratic problems [46]. However, mixed-norm problems have only recently attracted researchers' attention [28,47]. A widely used algorithm for solving minimization problems with *l_q_* norms for 1 ≤ *q* < 2 is the IRLS [46]. It is an iterative algorithm based on the approximation of a *l_q_* norm in a sequence of easily-solvable weighted least squares problems. The pseudocode of the UTSR algorithm based on IRLS is shown in Algorithm 1.


**Algorithm 1.** Pseudocode of UTSR algorithm using IRLS.
 UTSR(*v(u,t)*, **H**, *α, β, ∊*) **begin**  **v** ← vec [*υ*(*u, t*)]  f^(0)^ ← **H**^†^**v**  **foreach**
*j=0,1,2*,… **do**   
$W_{R}^{\left(j\right)}=\text{diag}\left(\frac{1}{\left|f_{m}^{\left(j\right)}\right|+\beta }\right)$   
$f^{\left(j+1\right)}\leftarrow \text{pcg}\left({\text{H}}^{†}\text{H}+\alpha^{2}W_{R}^{\left(j\right)},{\text{H}}^{†}v\right)$   **if** ‖**f**
^(^*^j^*^+1)^ − **f**^(^*^j^*^)^‖ ≤ *∊*
**then**    **f**_UTSR_ ← **f**^(^*^j^*^+1)^    **finished**  **end** **end**


The parameters *β* and *∊* are respectively a small constant to avoid zero division and an update tolerance used as stop criterion. We used the CG method [48] to solve the weighted least squares problem in each iteration of the IRLS (line 7 of Algorithm 1).

In other works that use regularization methods to reconstruct ultrasonic NDT images [14,39–41,49], the system model **H** is implemented as a true matrix. The drawback of this approach is the storage of large matrices, e.g., in a linear scanning with 30 *A-scan* signals, each having 500 samples, the **H** matrix has 15,000 × 15,000 elements. We have implemented **H** and **H**^†^ as *MATLAB^®^* functions, thus avoiding this inconvenience.

The choice of *α* is critical for a good reconstruction [35]. Whereas small values approximate a LS solution, which may be unstable, large values may oversmooth the solution due to excessive regularization. There are several methods to choose *α* such as visual inspection, the discrepancy principle, the L-curve criterion and generalized cross-validation [28,35]. In this work, we use the method described in Zibetti *et al.* [50] which is similar to the L-curve criterion. The *α* obtained was 0.0212.

## 4. Simulations

The UTSR algorithm is implemented in *MATLAB^®^*, being tested and compared with other four algorithms: *B-scan*, SAFT [14], *ω-k* SAFT [19] and RLS—with **L** = **I**. For this, an image of a point-like reflector is reconstructed from *A-scan* signals simulated by Equation (15). The parameters used in the model follow exactly the UIS specifications described in Section 2.1. *A-scan* signals are generated like signals acquired by the UIS (Section 2.2) with spatial sampling period of 1 mm. Gaussian white noise is added to *A-scan* signals with SNR of 20 dB. The simulation considers a point-like reflector inside a steel block (*c* = 5860 m/s). This point is located at *x* = 30 mm and *z* = 40 mm. The ROI is 15 mm ≤ *x* ≤ 45 mm and 18 mm ≤ *z* ≤ 58 mm. Both RLS and UTSR algorithms use *α* = 0.0212.

**Figure 3:**
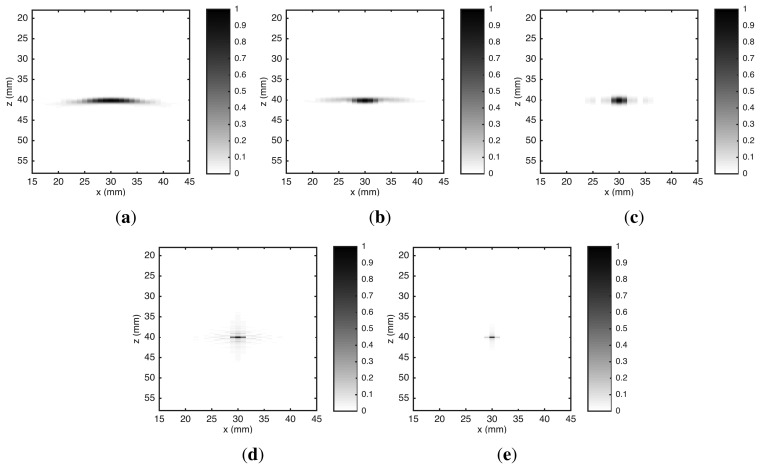
Reconstructed images of a point-like reflector from simulated data. The algorithms used in the reconstruction are: (**a**) *B-scan;* (**b**) SAFT; (**c**) *ω-k* SAFT; (**d**) RLS and (**e**) UTSR. Amplitude scale is normalized to maximum absolute value.

Figure 3 shows the images reconstructed by the five algorithms. The point-like reflector appeared at correct position of ROI in all images. However, the lateral spread—due to the diffraction effect—was clearly different in the five algorithms. The greatest spread occurred in the *B-scan* algorithm because it does not have any kill to compensate diffraction effects. In SAFT, the delay-and-sum (DAS) process carried out a partial compensation for diffraction effects, resulting in a reduction of lateral spread. The lateral spread was decreased by *ω-k* SAFT due to inclusion of a Wiener filter to compensate diffraction effects [19]. However, none of them presented a significant decrease in vertical spread. This occurred because they do not have any compensation for limited frequency response of the transducer. The spread produced by the RLS algorithm was smaller than the one by *ω-k* SAFT. However, this reduction occurred only in vertical spread. The inclusion of the transducer frequency response in the UIS model—used by the RLS algorithm—caused a compensation for limited transducer bandwidth. In the lateral direction, the compensation for diffraction effects in the RLS algorithm was equivalent to a Wiener filter [35]. The UTSR algorithm also uses the UIS model, therefore it also has compensation for diffraction effects and limited frequency response of the transducer. However, the spread was narrower, both laterally and vertically, because the *l*_1_ norm regularization has a tendency to prefer sparse solutions [44,45].

A quantitative way to assess reconstruction quality is through *array performance indicator* (API) [21]. API is a dimensionless measure of the spatial dimension of the spread of a point. It is defined by Holmes *et al.*, as:

(27) $$\text{API}=\frac{{\text{A}}_{50\%}}{\lambda^{2}}$$

where *A*_50%_ is the area within which the spread of a point is greater than a half from its maximum value and λ is the wavelength. Table 1 presents API values calculated on the images reconstructed by each algorithm. For comparison, the API value for the TFM image obtained by Holmes *et al.*—using simulated data—is included in Table 1.

If the B-scan image is taken as the baseline, API was reduced by 90.2% in the RLS image and by 95.6% in the UTSR image. Furthermore, the API for the UTSR image was reduced when compared with the API for the TFM image.

## 5. Experimental Validation

The experimental validation of the UTSR algorithm was carried out reconstructing images from *A-scan* signals acquired by the UIS described in Section 2.1. The UIS performed a linear scanning, by moving the contact transducer on the upper surface of specimens (plane X-Y), along the X axis. The spatial sampling period was 1 mm. The *A-scan* signals were collected from two different objects: (**I**) a steel block with a single side-drilled hole (SDH) and (**II**) a steel block with four SDHs at different depths.

### 5.1. First Object Reconstruction

**Figure 4:**
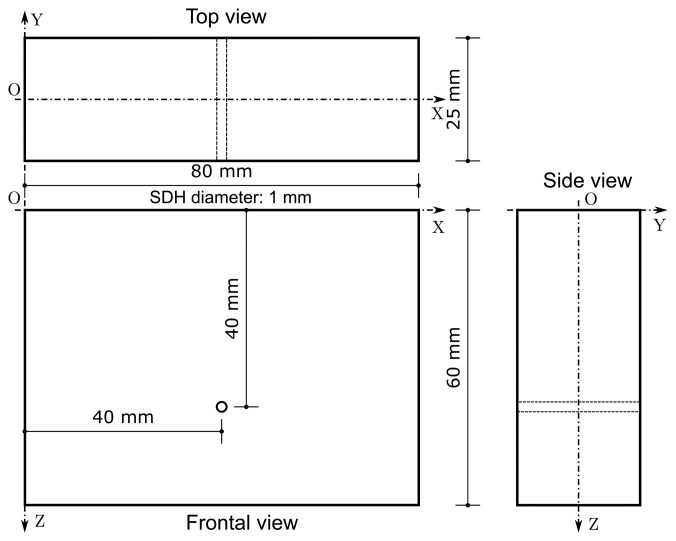
Geometry of the first inspected object. It was manufactured of steel and has a SDH with 1 mm of diameter, centered at *x* = 40 mm and *z* = *40* mm.

The first inspected object was a steel block with dimensions shown in Figure 4. The discontinuity in the object is a SDH with 1 mm of diameter, centered at position *x* = 40 mm and *z* = 40 mm. The ROI is 25 mm ≤ *x* ≤ 55 mm and 18 mm ≤ *z* ≤ 58 mm. Both RLS and UTSR algorithms used *α* = 0.0212.

**Figure 5:**
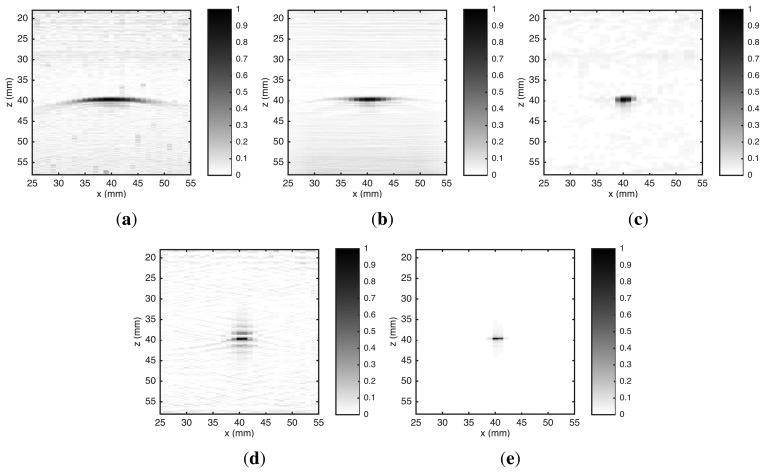
Reconstructed images of a SDH with 1 mm of diameter from real data acquired by UIS. The algorithms used in the reconstruction are: (**a**) *B-scan*; (**b**) SAFT; (**c**) *ω*-*k* SAFT; (**d**) RLS and (**e**) UTSR. Amplitude scale was normalized to maximum absolute value.

**Figure 6:**
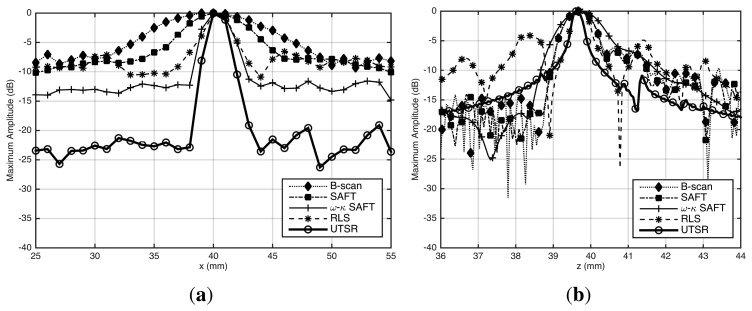
(**a**) Lateral and (**b**) depth profile graphs comparing maximum amplitudes (in dB) of reconstructed images for each image position on the X and Z axes. These profile graph shows SNR of reconstructed images by *B-scan*, SAFT, *ω-k* SAFT, RLS and UTSR algorithms.

Figure 5 shows images reconstructed by the five algorithms using data from the UIS. Those images show good consistency between the simulated and real data. This demonstrated that the UIS model is valid. We can see some vestigial noise in reconstructed images of Figure 5. Visually, the *ω-k* SAFT and the UTSR algorithms had reconstructed images with a higher SNR than others. Figure 6 presents the lateral (X axis) and depth (Z axis) profiles graphs showing maximum amplitudes—in dB—for each image. The lateral profile graph (Figure 6a) shows that images reconstructed by *B-scan*, SAFT and RLS algorithms had SNR about 7 dB. The image reconstructed by the *ω-k* SAFT algorithm had SNR about 12 dB. The SNR of reconstructed image by UTSR algorithm was about 19 dB. This noise reduction was also improved by the *l*_1_ norm regularization. The sparse solution has fewer nonzero elements, therefore image noise is noticed only around the SDH. The depth profile graph (Figure 6b) shows how narrower is the spread of a point for the UTSR reconstruction.

Table 2 shows the API calculated for the images of Figure 5 and the API for TFM image obtained by Holmes *et al.* with real data. Although the reflector used by Holmes *et al.* is different in relation to this work, the reflections caused by both are similar and a fair comparison is possible. As it can be seen, the experimentally obtained APIs were consistently higher than the simulated values. This is due to two issues: (**I**) the difference between the bandwidth of the simulated and experimental data caused by approximations in UIS model; and (**II**) the misalignment between the acquisition grid and the ROI. Nevertheless, the API value obtained for the UTSR algorithm was better than the API value for TFM algorithm obtained with data collected by FMC [21].

### 5.2. Second Object Reconstruction

**Figure 7:**
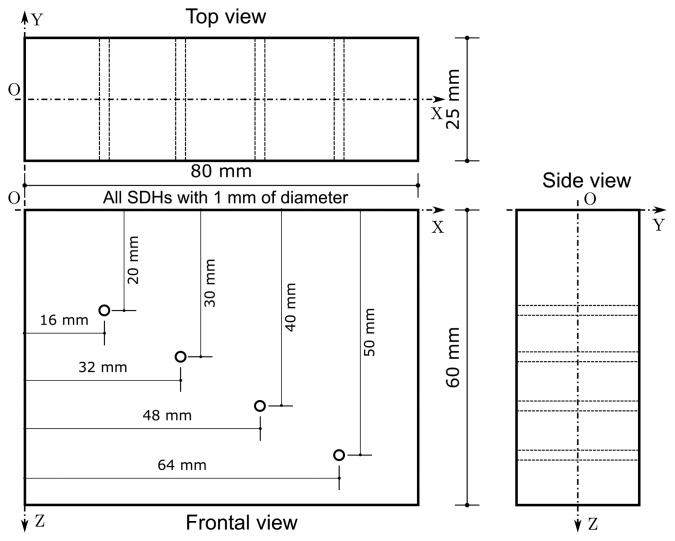
Geometry of the second inspected object. It was manufactured of steel and it has four SDHs with 1 mm of diameter centered at positions *x* ={16,32,48,64} mm and *z* = {20,30,40,50} mm.

The second object inspected was also a steel block with dimensions shown in Figure 7. This object has four SDHs with 1 mm of diameter, centered in positions *x* ={16,32,48,64} mm and *z* = {20,30,40,50} mm, respectively. The ROI is 3 mm ≤ *x* ≤ 77 mm and 18 mm ≤ *z* ≤ 58 mm. RLS and UTSR algorithms use both *α* = 0.0212.

**Figure 8:**
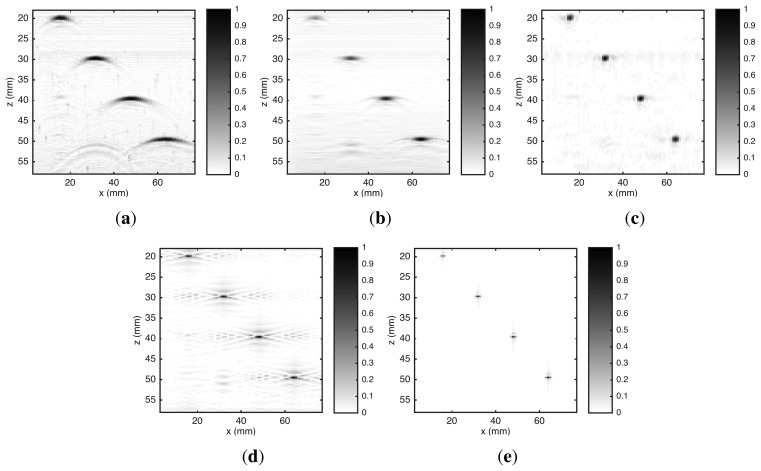
Reconstructed images of a steel block with four SDHs with 1 mm of diameter from real data acquired by the UIS. The algorithms used in the reconstruction are: (**a**) *B-scan*; (**b**) SAFT; (**c**) *ω-k* SAFT; (**d**) RLS and (**e**) UTSR. Amplitude scale was normalized to maximum absolute value.

Figure 8 presents five reconstructed images. As noted by Schmitz *et al.* [5], we can see in Figure 8a that the spreading of the points gets wider as depth increases. In all reconstruction algorithms, this problem was corrected. It is also visible in Figure 8a–d the occurrence of “artifacts” due to: (**I**) multiple reflections from discontinuities at shallow depths—20 mm—and (**II**) reflections of transversal waves generated on discontinuities by *mode conversion* in a solid medium [3]. However, due to the use of a sparsity-promoting norm in regularization term, these “artifacts” were eliminated in the reconstruction carried out by the UTSR algorithm.

Among the images of Figure 8, the reconstructed by *ω-k* SAFT algorithm (Figure 8c) is visually closer to the inspected object. However, this result is misleading. Only the incident waves that reflect on a small area on the surface of the SDH returns to the transducer. In a lateral cross section, this area approaches a point. Thus, the image reconstructed by UTSR algorithm (Figure 8e) represents better the acoustic reflectivity of the SDHs in the second object. In the image reconstructed by *ω-k* SAFT algorithm, the spreading of these points is wider.

## 6. Conclusions

This paper presented a new image reconstruction algorithm for ultrasonic NDT. *A-scan* signals acquired by an ultrasonic imaging system with a monostatic transducer in pulse-echo configuration were used. The proposed method, referred to as UTSR algorithm, is based on a regularized least squares problem resolution using the *l*_1_ norm in regularizer and **L** = **I**. This problem is solved with IRLS and CG algorithms.

Using API as quality metric, we assessed ability of the algorithms to reconstruct an image of a point-like reflector. The reconstruction quality of the UTSR algorithm was tested using both simulated and real data. Same input data were also used with four traditional ultrasonic imaging reconstruction algorithms: *B-scan*, SAFT, *ω-k* SAFT and RLS. Simulated data were generated by an analytical model of ultrasonic imaging system. This model was also used by both RLS and UTSR algorithms. Experimental validation was carried out using the reflection from a SHD with 1 mm of diameter—first object—and four SDHs with 1 mm of diameter at different depths—second object.

The proposed UTSR algorithm demonstrated a significant improvement in the reconstruction quality compared to the traditional algorithms. If the *B-scan* is taken as the baseline, the API was reduced from 4.08 to 0.18 using simulated data and from 5.38 to 0.47 using real data. Those API were also finer than presented by Holmes *et al.*, [21] for the TFM algorithm using FMC data from a UIS with a phased array transducer. The UTSR algorithm had also demonstrated an improvement in SNR of reconstructed images. These were due to (**I**) compensations of the diffraction effect and limited frequency response of ultrasound transducer, that are included in algorithm by the system model; and (**II**) the *l*_1_ norm in regularization term that it has a tendency to promote sparse solutions.

The UTSR algorithm demonstrated a great potential in finding small defects, mainly by resolution and SNR improvement of reconstructed images. An advantage of using this algorithm is that it can be applied to *A-scan* signals acquired by conventional UISs without the need of phased array transducers. However, its extension to phased array UISs is straightforward by simply modifying the system model. Future work are driving in this direction.

## Figures and Tables

**Table 1:** API for images reconstructed from simulated data. Point-like reflector at *z* = 40 mm.

**Algorithm**	**API**	**Difference from *B-scan***
B-scan	4.08	0%
SAFT	2.09	−48.8%
*ω-k* SAFT	1.81	−55.6%
RLS	0.40	−90.2%
UTSR proposed	0.18	−95.6%
TFM [21]	0.46	−88.7%

**Table 2:** API for images reconstructed from experimental data. SDH geometrical center at *z* = 40 mm.

**Algorithm**	**API**	**Difference from *B-scan***
*B-scan*	5.38	0%
SAFT	3.16	−41.3%
*ω-k* SAFT	1.92	−64.3%
RLS	0.81	−84.9%
UTSR proposed	0.47	−91.3%
TFM [21]	0.77	−85.7%
